# A Case of Persistent Foot Pain in a Neurofibromatosis Type I Patient

**DOI:** 10.1155/2012/479632

**Published:** 2012-01-18

**Authors:** Vasilis Stavrinides, Salim Nasra

**Affiliations:** ^1^Department of Neurosurgery, John Radcliffe Hospital, Headington, Oxford OX3 9DU, UK; ^2^Department of Trauma and Orthopedics, St Mary's Hospital, Newport, Isle of Wight PO30 5TG, UK

## Abstract

*Introduction*. This is the case of a young male patient who presented to his family physician with atypical left foot pain, which was extremely resistant to analgesia and caused significant disability. Despite extensive investigations, the cause of his pain was not identified until 18 months after his initial symptoms, when the official diagnosis of malignant peripheral nerve sheath tumour (MPNST) was made. Detailed review of the patient's past history established the diagnosis of type I neurofibromatosis (NF-1), previously undetected. *Discussion*. NF-1 is an autosomal dominant genetic disorder caused by loss of function mutations of the *NF1* gene in chromosome 17. Patients with this condition are at increased risk for developing MPNSTs which, however, are treatable only in early stages. *Conclusion*. Although monitoring NF-1 patients for the development of MPNSTs is common practice, the index of clinical suspicion in patients without an established NF-1 diagnosis is low. Any atypical pain in young adults should raise the possibility of this malignancy, and this case illustrates the fact that MPNSTs can be the first manifestation of NF-1 in patients previously undiagnosed with the disease.

## 1. Case Report

A 24-year-old male smoker presented to his GP with a 6-month history of right foot pain extending from the base of the first toe to the plantar surface of the foot. The pain was fluctuating in severity, did not correlate with specific activities, and was only partially relieved with common analgesics. The patient's work involved prolonged standing and heavy lifting. He had had bilateral pes planus and valgus heels during his childhood but had been asymptomatic for several years. Two abdominal nodules had been excised by a dermatologist two years prior to the onset of his presenting complaint. The histopathology report confirmed they were a fibroma and a leiomyoma of no particular clinical significance. Apart from a few other minor injuries, his past medical and surgical history was otherwise unremarkable.

The pain was attributed to the patient's previous pes planus deformity and was treated conservatively with common analgesia by the GP. Over the next three months the pain did not resolve and the patient was referred to the local orthopedics outpatient clinic. Extensive X-ray studies and blood tests including inflammatory and immunological markers were all normal, and the patient was referred to Orthotics for foot arch support.

Despite the arch support, the pain progressively worsened over the next 6 months. A foot MRI revealed mild, early degenerative 1st MTP joint changes with a small amount of fusion, but no other abnormalities. The diagnosis of reflex sympathetic dystrophy was also considered and supported by the findings of a radionuclide isotope scan. Although rocker soles and stronger analgesia (including oral morphine) were suggested, these failed to control the symptoms. Although this was followed by physiotherapy and multiple guanethidine blocks, symptoms remained uncontrolled and caused significant disability 18 months after the patient's first GP visit.

**Figure 1:**
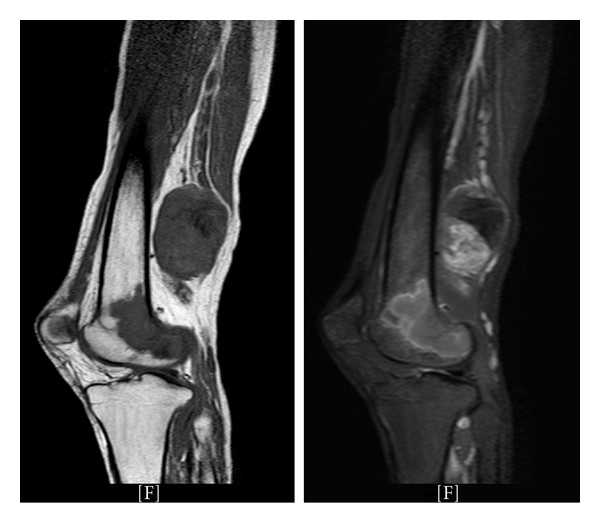
Urgent MRI of the right lower limb; T1 weighted (left), T2 weighted (right). A large (approx. 15 cm), oval, smoothly defined heterogenous mass with a large necrotic centre is clearly visible behind the lower femur (white arrows). There is also an area of altered signal within the femur itself involving the condyle, suggestive of bony metastasis (white arrowheads). The likely diagnosis is malignant peripheral nerve sheath tumor (MPNST) with a differential diagnosis of lymphoma or other soft tissue sarcoma. The character and distribution of the pain could signify a sciatic nerve origin.

In the following weeks the patient developed progressive weight loss, fatigue, and muscle wasting below the knee. He noticed for the first time a firm round mass (approximately 15 cm) on the lower aspect of the posterior thigh. He was referred back to Orthopaedics and an urgent MRI confirmed an oval, smoothly defined heterogenous mass in addition to signal changes in both right and left femurs, most likely representing metastatic foci (Figure 1).

The appearances were most suggestive of metastatic malignant peripheral nerve sheath tumour (MPNST), and the patient was urgently referred to Oncology. Chest CT and whole-body MRI revealed the presence of multiple lung and bone metastases, consistent with stage IVb malignant disease. Predisposing factors for this rare malignancy including NF-1 were considered. A detailed clinical examination revealed freckling of the right axillary region. In addition, the histology slides from the patient's abdominal skin nodule biopsy were reexamined by a specialist. The initial fibroma diagnosis was inconsistent with histopathological findings, which were more in keeping with an intraneural neurofibroma, part of a plexiform neurofibroma.

Based on the National Institute of Health (NIH) diagnostic criteria, the patient was diagnosed with neurofibromatosis type I [1]. The patient was started on palliative chemotherapy with doxorubicin, but unfortunately died 4 months later, almost 24 months from his initial complaint.

## 2. Discussion

Type I neurofibromatosis (NF-1) is a common autosomal dominant neurocutaneous disorder, characterized by multiple café au lait spots, axillary and inguinal freckling, multiple cutaneous neurofibromas, and iris Lisch nodules [2]. Clinical diagnosis of NF-1 is made using the 1988 NIH diagnostic criteria presented in Table 1.

NF-1 is caused by loss of function mutations in the *NF1* gene in 17q11.2. This leads to defective production of neurofibromin, a guanosine triphosphatase-activating 

protein that helps maintain the protooncogene Ras in its inactive form [3]. Loss of neurofibromin predisposes to increased tumorigenesis, and malignant disease can appear in either childhood or adulthood, with malignant peripheral nerve sheath tumours (MPNSTs) being most common [4]. Several pathways are thought to be involved in the development of tumours associated with NF1: rat sarcoma viral oncogene homologue (RAS)-mitogen-activated protein kinase (MAPK), mammalian target of rapamycin (mTOR), and P21 protein (Cdc42/Rac)-activated kinase 1 (PAK1) [5, 6].

NF-1 patients have a lifetime risk of 8–13% to develop MPNSTs, which are the leading cause of NF1-related mortality. In current clinical practice the diagnosis of MPNST should always be considered in NF-1 patients, especially those with persistent pain (that lasts over 1 month or disturbs sleep), new neurological deficits, or alteration in the characteristics of a known neurofibroma [7]. Surgical resection is the mainstay of treatment; however, because of increased metastatic potential and resistance to chemotherapy and radiation the prognosis is poor. Five-year survival rates still only reach 20–50%, despite latest efforts to identify potential molecular targets [8].

MPNSTs should be included in the differential diagnosis of persistent or atypical pain in a young adult. Although rare, these malignancies are commonly associated with NF-1 and can be the first manifestation of the disease, as this case illustrates. Early diagnosis is paramount as survival rates are extremely poor in advanced stages of the malignancy.

## 3. Conclusion

MPNSTs can present with atypical symptoms in the young adult population and are often difficult to diagnose in previously healthy individuals. Unexplained pain that causes significant disability despite strong analgesia should be thoroughly investigated until serious underlying malignancy is excluded. Although not exclusively associated with NF-1, detection of an MPNST should prompt the physician to consider the diagnosis and seek specialist help.

## Figures and Tables

**Table 1:** National Institute of Health diagnostic criteria for neurofibromatosis type I [1]. The criteria are met in an individual if two or more of the features listed are present.

Diagnosis of neurofibromatosis type 1 (NF1)	
(1) Six or more café au lait macules >5 mm in greatest diameter in prepubertal individuals and >15 mm in greatest diameter in postpubertal individuals	
(2) Two or more neurofibromas of any type or one plexiform neurofibroma	
(3) Freckling in the axillary or inguinal regions (Crowe's sign)	
(4) Optic glioma	
(5) Two or more Lisch nodules (iris hamartomas)	
(6) A distinctive osseous lesion such as sphenoid dysplasia or thinning of long bone cortex with or without pseudoarthrosis	
(7) A first-degree relative (parent, sibling, or offspring) with NF1 by the above criteria	
